# Phase evolution and structural modulation during in situ lithiation of MoS_2_, WS_2_ and graphite in TEM

**DOI:** 10.1038/s41598-021-88395-1

**Published:** 2021-04-27

**Authors:** Chanchal Ghosh, Manish Kumar Singh, Shayani Parida, Matthew T. Janish, Arthur Dobley, Avinash M. Dongare, C. Barry Carter

**Affiliations:** 1 Department of Materials Science and Engineering, University of Connecticut, Storrs, CT 06269 USA; 2 EaglePicher Technologies, East Greenwich, RI 02818 USA; 3 Department of Chemical and Biomolecular Engineering, University of Connecticut, Storrs, CT, 06269 USA; 4 Center for Integrated Nanotechnologies (CINT), Sandia National Laboratories, Albuquerque, NM, 87185 USA

**Keywords:** Materials for energy and catalysis, Nanoscale materials

## Abstract

Li-ion batteries function by Li intercalating into and through the layered electrode materials. Intercalation is a solid-state interaction resulting in the formation of new phases. The new observations presented here reveal that at the nanoscale the intercalation mechanism is fundamentally different from the existing models and is actually driven by nonuniform phase distributions rather than the localized Li concentration: the lithiation process is a ‘distribution-dependent’ phenomena. Direct structure imaging of 2H and 1T dual-phase microstructures in lithiated MoS_2_ and WS_2_ along with the localized chemical segregation has been demonstrated in the current study. Li, a perennial challenge for the TEM, is detected and imaged using a low-dose, direct-electron detection camera on an aberration-corrected TEM and confirmed by image simulation. This study shows the presence of fully lithiated nanoscale domains of 2D host matrix in the vicinity of Li-lean regions. This confirms the nanoscale phase formation followed by Oswald ripening, where the less-stable smaller domains dissolves at the expense of the larger and more stable phases.

## Introduction

In the last few decades intercalation science has gained enormous interest due to its widespread application in energy storage materials^1–3^. With the advent of intercalation technology, the alkali-ion batteries (lithium-ion batteries—LIBs, sodium-ion batteries—NIBs) became an integral part of everyday life^4^. Research is in progress in the quest for superior electrode materials for improved charging–discharging behavior with higher safety^5–10^. The search for alternate electrode materials commenced dates back to the late 1960s with a significant amount of research on the electrical, optical, and structural properties of a series of layered transition metal dichalcogenides^11,12^. Carter and Williams provided direct evidence of the formation of ordered superlattice structures in NbSe_2_ and MoS_2_ with sodium intercalation through transmission electron microscopy (TEM)^13^. Comprehensive reviews on the chemistry of the intercalated chalcogenide hosts and also on the cathode materials of Li batteries during the early 1970s to the beginning of the current century have been well documented in the literature^14,15^. In recent years, transition metal dichalcogenides (TMDs), especially the sulfides, have emerged as the most preferred alternatives electrode material for the alkali-ion batteries^16–21^.

During the intercalation process, the alkali ions interact with the host matrix leading to significant changes in the microstructural and microchemical nature of the layered materials^22–25^. Understanding of the phase and structural changes in the intercalated materials is indispensable in order to tailor the improved host materials. Transmission electron microscopy (TEM) and its associated techniques have been extensively used to study the modified structural and chemical nature of the intercalated matrices^26,27^. However, in most cases, structural and chemical analysis has been carried out in the post-reacted specimen, where the finer details of the reactions and the dynamics of the structural transformations are lacking. It is always advantageous to record the intercalation process as it occurs so that the recording can then be analyzed for structural changes prior to and after the reaction process^27–29^.

Reports are available in the literature database of in situ studies of the intercalation process using diffraction-based techniques, e.g., XRD and neutron diffraction^30,31^. However, in situ interactions between the alkali ions and layered materials inside the TEM is the only technique that allows the direct visualization of the structural changes as well as associated morphological changes in *real time*^32–35^. In situ studies of lithiation and de-lithiation of 2D materials using an electrochemical liquid cell in the TEM is one of the most established TEM based technique in this regard and is more likely to mimic the *operando* behavior of the electrochemical storage cell^36–38^. More recently, with the development of more sophisticated and improvised TEM holders, direct visualization of the solid-state reactions between the alkali ions and the layered systems with the application of external electrical biasing inside the TEM also becomes possible at atomic resolution^27,39–41^. The intercalation mechanism is, in-principle, different for solid electrolyte cells and liquid electrolyte cells. With liquid electrolyte cells, the electrodes are fully dipped into the electrolytes, prompting the full infiltration of the liquid into the electrodes. This dipping further promotes different chemical interactions such as, conversion and alloying of all active materials in the electrodes along with the Li intercalation. The consumption of the Li ions at the solid electrolyte interphase (SEI) further reduces the charge carriers at the electrolytes and subsequently increases the ion-transfer resistance^42^. For solid electrolytes, as in the present study, the Li ions intercalate into the vdW gaps of the host matrix with the application of an external electrical bias and subsequently de-lithiate with the removal of the external bias. Thus, the movement of the Li ions in the host matrix produces the current.

The intercalation mechanism in the TMDCs is expected to differ from the present generation of carbon-based materials mainly due to its relatively large van der Waals gap, which enables them to accommodate a higher concentration of Li without significant change in the relative layer dimensions. Moreover, the nature of the lithiation also varies among the TMDs itself, which can be attributed to the nature of the binding energies between Li with the respective 2D-MX_2_ (M = Mo, W, etc.; X = S) and also to the interactions between the Li-ion and the chalcogen atoms. The insertion of the intercalating ions in between the MoS_2_ layers can also be associated with the structural transformation from the 2H to 1T phase where the metal-coordination changes from prismatic to octahedral^43–47^.

It has already been reported that above an incorporation of 30–40% Li, a structural re-orientation take place in monolayer MoS_2_, resulting in the transformation from 2H to 1T phase^48,49^. This type of transformation is associated with the accumulation of localized strains and can lead to the formation of lattice defects. For Li intercalation into the MoS_2_ when viewed in the edge-on condition, the formation of lattice defects has been already reported during in situ TEM studies; the lattice defects form thin bands comprising 5–7 atomic planes as observed using HRTEM imaging^40^. A similar phenomenon has also been reported during Na intercalation in MoS_2_, where the formation of the crystal defects and large density of interlayers fragmentation is explained in the light of a large amount of in-plane misfit stresses due to the concentration gradient of intercalant during sodiation/de-sodiation^41,50^. Density functional theory (DFT) simulations can have a major role in the understanding of the associated structural transformations^51^ where the recent analyses show the presence of lattice defects including vacancies, antisite defects, and grain boundaries enhance the probability of the Li absorption making defective monolayer MoS_2_ as a suitable anode material for LIBs and NIBs^41,52–57^. In a recent study from the authors, it is reported that the intercalation associated 2H-1T phase transformation in MoS_2_ is essentially a localized phenomenon leading to a vertically stacked dual-phase microstructure^58^.

Understanding structural modifications and associated chemical changes due to Li intercalation in the MoS_2_ and WS_2_ matrices are central to uncover the underlying reaction mechanism and thereby enables to gauge their behavior as an energy storage material in comparison with graphite. Several phases have been observed with the introduction of Li ions with 2H-MoS_2_ and WS_2_ flakes^59–62^. The Li^+^ changes the local symmetry of 2H phases of MoS_2_ and WS_2_ by reacting with S atoms in favor of 1T-Li_x_MoS_2_ and 1T-Li_x_WS_2_ phases, as demonstrated both experimentally and by computer modeling^63–66^. After the 2H-to-1T phase transformation, the continued reaction can lead to intensified conversion resulting in fragmentation of MoS_2_ into LiS_2_ and Mo nanoparticles^67^. WS_2_ nanoflakes after complete lithiation were found to be converted into Li_2_S and W nanoparticles^68^.

The major motivation of this study is to understand the mechanism of Li intercalation in sulfide-based TMDs with the aid of atomic-resolution TEM and DFT modeling and to compare these results with an analysis of the existing models of the lithiation mechanism in graphite. The current understanding of the lithiation mechanism and the formation of intermediated structures in graphite is based on the two classic models, namely the Rüdorff-Hofmann model^69^ and the Daumus–Herold model^70^. The Rüdorff–Hofmann model considers the layer-by-layer intercalation of each van der Waals gap and separated by layers that do not have intercalated Li, which results in the formation of the partially lithiated phases, e.g., LiC_18_ and LiC_12_. For example, in LiC_18_, every third interlayer spacing would be lithiated, while in LiC_12_, every second interlayer spacing would hold Li. The Daumus–Herold model proposes the partial filling of each of the individual interlayers leading to the formation of partially lithiated domains that subsequently grow as more Li intercalates into the system and eventually leads to the formation of the LiC_6_ phase. Both of these models assume that the formation of the LiC_x_ depends on the Li concentration in its nearest neighborhood of the C atoms.

Direct microscopic evidence of this phenomenon in *real time* is relatively scarce and hence that the formation of the partially intercalated LiC_x_ and its dependence over the distribution of Li in the localized domains is also not well understood. The reaction mechanism is expected to differ in TMDs, owing to the fundamental differences in their structure from graphite; in the TMDs the vdW gap is between the chalcogen atoms, but observations imply that diffusion through other layers is possible. Moreover, the intercalation pathway can also vary among the TMDs itself, depending on the nature of metal–chalcogen bonds. Furthermore, the interaction between the intercalants and the chalcogen atoms can also dictate the mechanism in each of the TMDs differently. It has been reported that despite the larger interlayer separation in WS_2_ compared to MoS_2_, a greater quantity of Li intercalates in MoS_2_ in similar intercalating conditions^62^. The rationale behind this difference in Li intercalation in the two materials is not yet understood and demands in-depth studies combining electron microscopy and modeling.

The purpose of the present communication is to study the phase evolution, structural modification and chemical changes in mechanically exfoliated MoS_2_ and WS_2_ flakes during in situ solid-state reactions with Li inside TEM and further compared with that of the intercalated graphite lithiated at similar experimental conditions. The transformed structures at the atomic scale of lithiated phases are evaluated with the energetics of structures predicted using atomic-scale simulations based on density functional theory (DFT) and TEM image simulations using DFT-predicted microstructures. In a similar approach, in situ Li intercalation has been carried out with WS_2_ where the HRTEM results confirm the disorder in the pristine flakes after intercalation, together with the defect formation parallel to the c-axis. These results are also compared with the intercalated graphite, where the lattice expansion and the formation of LiC_6_ structure has been deduced. The feasibility of imaging the Li between the graphitic planes has been initiated using low-dose imaging with an aberration-corrected TEM.

## Results and discussion

**Figure 1:**
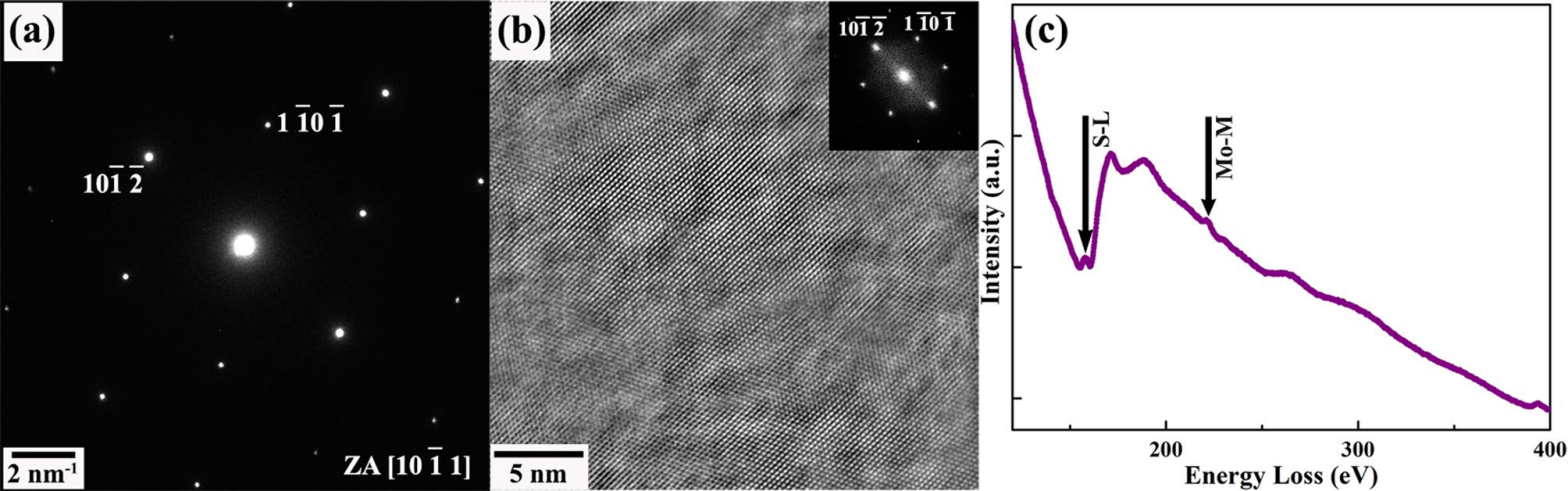
(**a**) Selected area electron diffraction pattern, (**b**) atomic resolution phase contrast micrograph and (**c**) electron energy-loss profile from a typical region of MoS_2_ in pristine state. Reflections correspond to two different lattice planes are marked in the SAD pattern (**a**). Power spectrum as generated from the HRTEM micrograph is shown as inset in (**b**).

A model of the exfoliated MoS_2_ specimen in its pristine state, before the reaction, is given in Fig. 1. The selected area electron diffraction pattern (SADP) as shown in Fig. 1(a) confirms the specimen has been oriented along [1 0 $\overline{1 }$ 1] zone axis during imaging. Additional spots pertaining to the double diffraction are also present in the SADP and is quite expected for a mechanically exfoliated 2D-materials where the presence of more than one layer is highly possible. Detailed indexing scheme of this diffraction pattern using Miller–Bravais indices^71,72^ is given in S4 (Supplementary material). This is further supported by the high-resolution TEM (HRTEM) micrograph and the corresponding power spectrum (inset) depicted in Fig. 1(b). Additionally, the atomic-resolution TEM image confirms the structure of the specimen as 2H-MoS_2_ and is essential as a reference to compare the phase after interaction with Li. Electron energy-loss spectra from the exfoliated MoS_2_ specimen is presented in Fig. 1(c) where the energy loss edges correspond to S-L and Mo-M are marked at 165 eV and 227 eV, respectively. The relative quantification of Mo and S are determined from the EELS profile after a background subtraction with power-law method, confirming the stoichiometry of the pristine specimen as MoS_2_.

**Figure 2:**
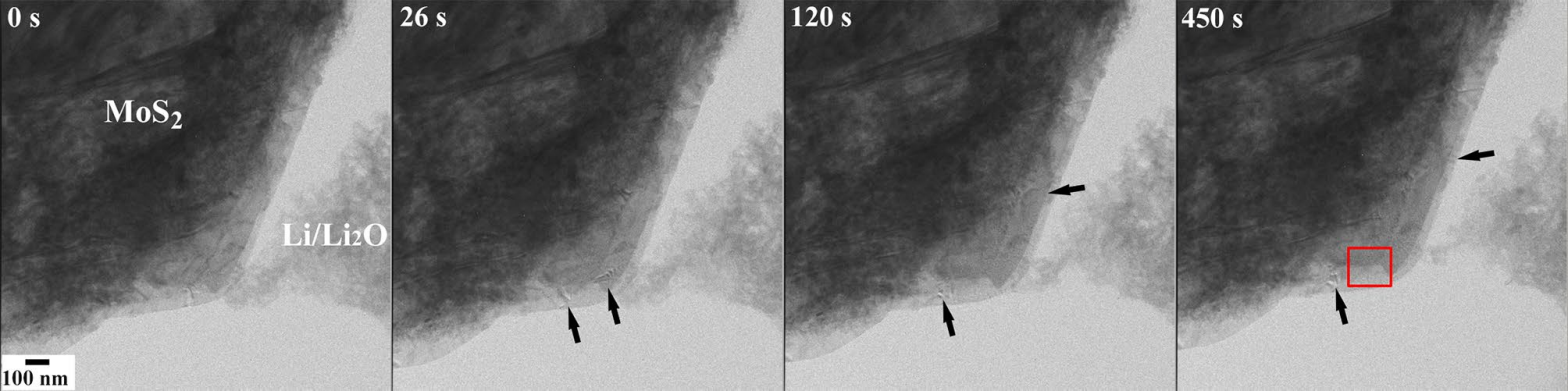
Snapshots at different time frames showing the microstructural changes during Li and MoS_2_ reaction. The changes are highlighted with arrows. Red box indicates the region for further investigation.

The microstructural evolution of the MoS_2_ flake during the reaction with Li has been studied in situ using the TEM. Selected snapshots at different time frames are displayed in Fig. 2. Post-lithiated specimen region subjected to subsequent microstructural and microchemical analyses are marked with red colored box. The recorded video of the in situlithiation process is given as a supplementary file (S1). During the lithiation process, the changes in the microstructural features are shown with arrows in the images. Moreover, as observed in the video, these changes in the microstructural contrast are associated with the movement of periodic bright and dark fringes. The changes in the image are predominantly due to strain-induced contrast and manifest themselves in the movement of bright and dark contours in the diffraction-contrast images. The presence of strain during the in situ reaction is not surprising and can be associated to the incorporation and movement of Li ions in between the van der Waals gaps of the layers. Localized foil deformation can also be caused by the mechanical disturbances during the physical contact of the Li to the thin exfoliated samples. However, mechanical disturbance is only visible at the beginning of the experimental set-up inside the TEM and will not affect the contrast once the reaction begins with the application of external electrical biasing. Some drift is observed in the in-situ reaction video even though it is recorded at a relatively low magnification. This is primarily because of the applied bias-current which continuously changes the reaction front, and the mechanical vibration induced during the physical contact between the Li metal and the host matrix. At very high magnification, in the domain of atomic-resolution imaging, this associated drift can cause the region of interest (ROI) to move out of the recording camera frame in a very short time. The specimen regions where the lithiation is intended to be carried out needs to be electron transparent and in suitable crystallographic orientation and to confirm this the specimen has been observed in the TEM prior to the lithiation (cf. Fig. 1). Similarly, the point of contact between the host and the Li/Li_2_O is also maintained in such a manner that the microstructural changes during the in-situ lithiation are clearly observable in TEM without influence from the specimen and probe thickness.

**Figure 3:**
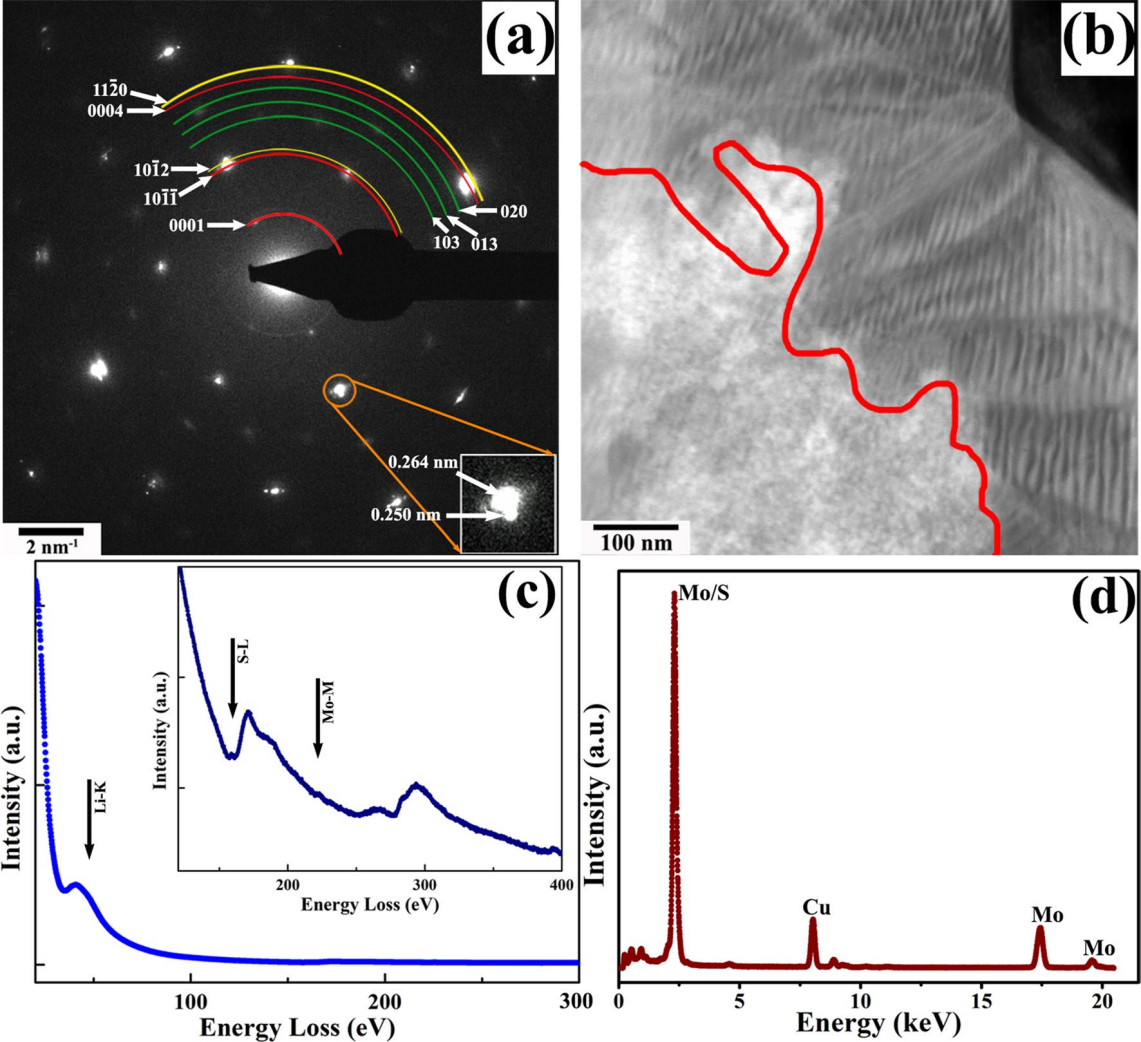
(**a**) SADP exhibiting the presence of newer phases in post-reacted MoS_2_. Reflections correspond to MoS_2_, Li_x_MoS_2_ and Li_2_S are depicted in yellow, red and green arcs, respectively along with their indices. Splitting in one of the MoS_2_ principal reflection is shown as inset. (**b**) HAADF image showing the reaction front in post-reacted specimen as portrayed in red boundary. (**c**) EELS profile from the post-lithiated specimen displaying the Li-K energy-loss edge. Corresponding Mo-M and S-L edges are given in inset. (**d**) XEDS profile displaying the Mo and S peaks.

Microstructural and microchemical changes in the MoS_2_ specimen after interacting with Li are shown in Fig. 3. A selected-area diffraction pattern (SADP) from the post-lithiated specimen is shown in Fig. 3(a); the presence of additional diffraction spots can be observed displaced from the principal reflections of MoS_2_. The SADP was recorded from the immediate peripheral of the Li-MoS_2_ junction where the solid-state interaction is a maximum. It is interesting to note that the principal spots of the MoS_2_ phase show a certain amount of splitting; a magnified view of one such split spot is shown as the inset of Fig. 3(a). The interplanar spacing from these split spots are measured as 0.264 nm and 0.250 nm, which can be assigned to Li_x_MoS_2_ and MoS_2_ structures, respectively. For superior visualization and representation of the newly appeared phases, colored concentric circular sections are used, where red and yellow colored arcs are representing Li_x_MoS_2_ and MoS_2_, respectively and reflections from Li_2_S (ICDD # 01-070-5970) is marked in green. Few typical diffracting planes from each of the phases are indexed accordingly. This observation essentially signifies the reduction of MoS_2_ into Mo and S. In order to diffract electrons, the reduced Mo should form an ordered crystal structure in significant amount. Non-appeareance of the electron diffraction spots signifies the absence of elemental Mo crystals in the marrix. However, the formation of amorphous Mo can not be ruled out. The presence of XEDS and EELS signal from nanoscale elemental Mo distributed over a MoS_2_ matrix is also not definite in this regard. During the lithiation reaction the intercalant traverses inside the MoS_2_ with the application of external biasing as displayed in the STEM-HAADF micrograph in Fig. 3(b). The reaction front is marked in red where the contrast difference is quite viable along both side of the boundary. This observation supports the general understanding of contrast distribution in a STEM-HAADF micrograph where regions with a lower average atomic-number would appear darker than the unreacted MoS_2_. The alternate fringe contrast as observed in the reacted region is predominantly the strain-induced contrast originated due to the intercalation of Li in between the MoS_2_ layers. Microchemical nature of the post-lithiated specimen was determined using EELS in TEM mode and one such EELS profile from the reacted region is shown in Fig. 3(c). The presence of the Li-K energy-loss edge is shown at 55 eV whereas the energy-loss peaks corresponding to S-L and Mo-M in the gain enhanced EELS profile is shown as inset. Elemental quantification of the energy-loss edges followed by the background subtraction after power-law curve fitting measure a relative amount of 0.6 Li in respect to MoS_2_ in the lithiated products. XEDS profile from the same region is displayed in Fig. 3(d) where the characteristic X-ray peaks of Mo and S can be observed. The observed Cu peak is from the TEM grid.

**Figure 4:**
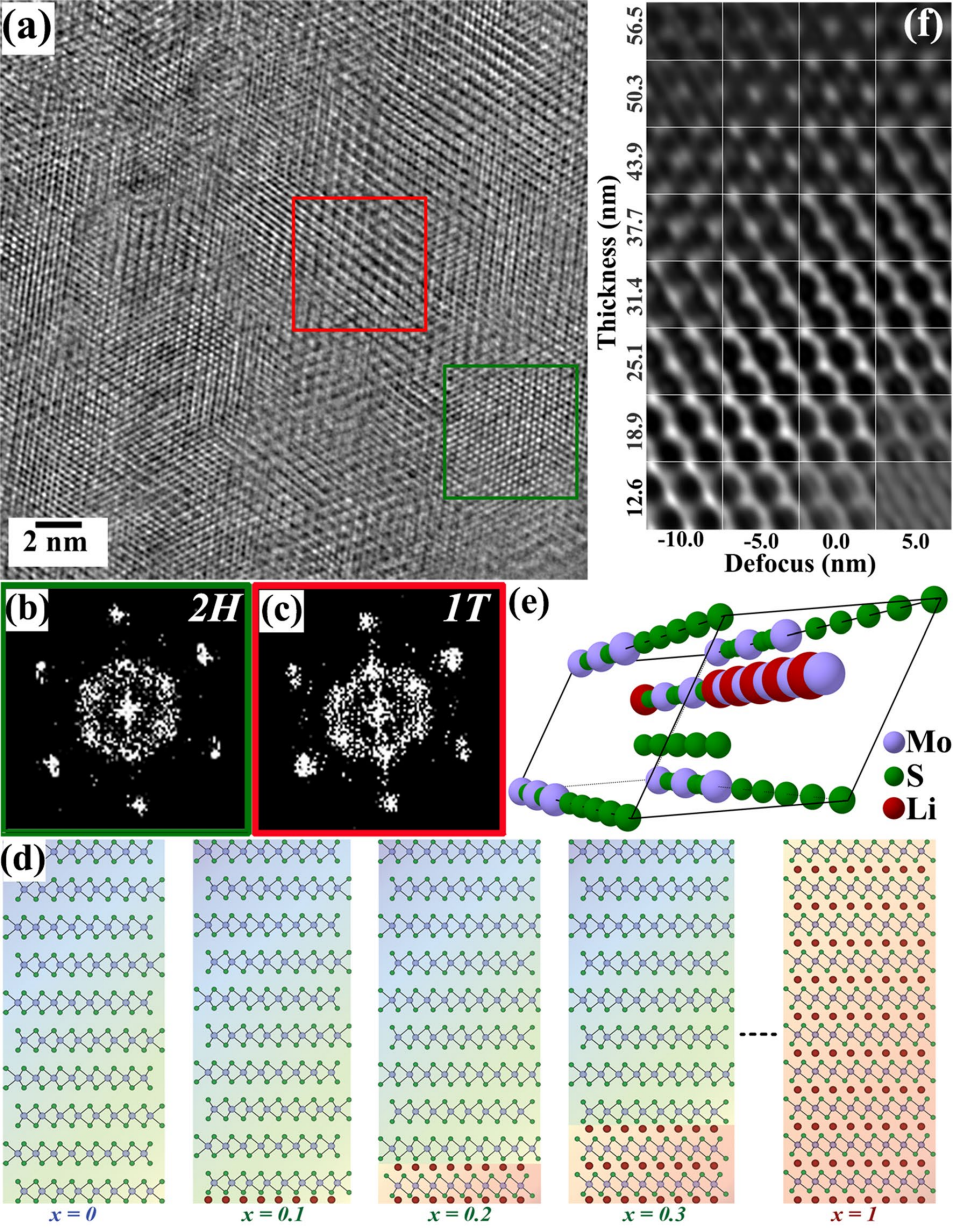
(**a**) A representative HRTEM micrograph from the post-lithiated MoS_2_ specimen displaying the domains 1T (red square) and 2H (green square) structures. The corresponding power spectra are shown in (**b**) and (**c**), respectively. (**d**) Schematic of Li intercalation in MoS_2_ based on the DFT modelling. Li, Mo, and S atoms are represented in wine, purple, and green colors, respectively. (**e**) Atomic structure projection of Li0.6-MoS_2_ along < 10 $\overline{1 }$ 1 > azimuth. (**f**) Thickness-defocus map of Li0.6-MoS_2_ phase along [10 $\overline{1 }$ 1] zone axis using multislice simulation.

Localized structural modulation and phase formation in the post-lithiated MoS_2_ have been studied using an image aberration-corrected TEM and the atomic-resolution phase-contrast micrograph of the same is depicted in Fig. 4(a). The localized structure modulation is quite obvious in the micrograph and for further analysis of the structural details two typical regions were selected. The power spectra as generated from these two regions are given in Fig. 4(b,c) and marked with the similar colored boxes as in the original micrograph. A detailed analysis of the power spectra confirmed the structures of the green and red squared regions as 2H and 1T phase of MoS_2_ and Li_x_MoS_2_, respectively. The microstructural features of the HRTEM micrograph indicate partial intercalation in the present situation: not all the octahedral and the tetrahedral sites in the MoS_2_ are occupied by Li.

To mimic the TEM observed structures, DFT simulations have been carried out to generate the partially lithiated structures for varying concentrations of Li. Schematics displaying the DFT calculated structures of partially lithiated MoS_2_ phases are given in Fig. 4(d). The structures are created using a 10-layer supercell of MoS_2_ with varying amounts of intercalated Li in between the van der Waals gap. The system was made to relax energetically (till forces on atoms reached less than 0.01 eV/Å) for each of the partially intercalated structures and the optimized crystallographic information files (cif) were extracted. Based on the elemental quantification as obtained from EELS studies, the optimized structure of Li_0.6_MoS_2_ has been considered for further processes. The perspective view of the Li_0.6_MoS_2_ unit cell structure as generated using the DFT optimized cif is shown Fig. 4(e). Subsequently, multislice image simulations have been carried out to generate the thickness-defocus map of the same structure along [10 $\overline{1 }$ 1] direction with the similar imaging conditions in which the micrograph Fig. 4(a) was recorded in aberration-corrected TEM at 0 nm defocus and shown in Fig. 4(f). The thickness of the specimen has been estimated using the t/λ calculation from the EELS profile. However, determination of the exact thickness from the 5 nm × 5 nm ROI is quite critical and considering the probable errors in the measurement, a thickness range of 20–40 nm is considered appropriate for the computational analysis.

The purpose of this excercise is to compare the lattice contrast of the simulated images to those with the ROIs displayed as red and green squares in Fig. 4(a). The linear contrast as shown in the simulated image is more closely resembles with the contrast from the red ROI, confirming the change of the structure due to Li intercalation. Whereas the ROI displayed in the green square provides atomic contrast, a comparison with the simulated image confirms that this region does not undergo any structural transformation due to the intercalation.

In the present study, in order to minimize beam damage, EELS is performed on a relatively large area and the relative concentrations of Li, Mo and S are averaged out over the entire area under consideration in addition to averaging over the thickness. The Li concentration may thus be averaged over several localized domains giving the average value as measured in EELS. It is non-trivial to probe and measure the Li concentration in the nm sized domains, because under the very localized electron beam the Li no longer remains stationary. The present imaging conditions consider the planar geometry of the lithiated phases from a multilayered MoS_2_ flake and it is likely that the intercalated Li concentration varies in each layer along the thickness of the flake. An uneven distribution of Li among the layers may result in an incomplete 2H-1T transformation in every individual layer. The HRTEM images recorded for the planar geometry may not be from a completely transformed 1T or 2H stack of layers, but rather from a hybrid 2H-1T mixed stack of layers. The completely transformed 1T or 2H structures would be more feasible during the intercalation of a monolayer MoS_2_. A 10-layer hybrid structure of 2H-1T phases predicted using DFT simulations is considered in this computational analysis of the lithiated phases. Multislice simulations using the DFT-optimized, lithiated MoS_2_ confirm that even with 0.1 relative concentration of Li atoms in MoS_2_, individual atomic contrast is no longer visible in the simulated HRTEM image; instead a linear contrast appears for both lithiated 1T or 2H phase (cf. figure S5 in supplementary material). These calculations thus further confirm that the 2H domain in Fig. 4(a) corresponds to the untransformed pristine MoS_2_^16,73^.

**Figure 5:**
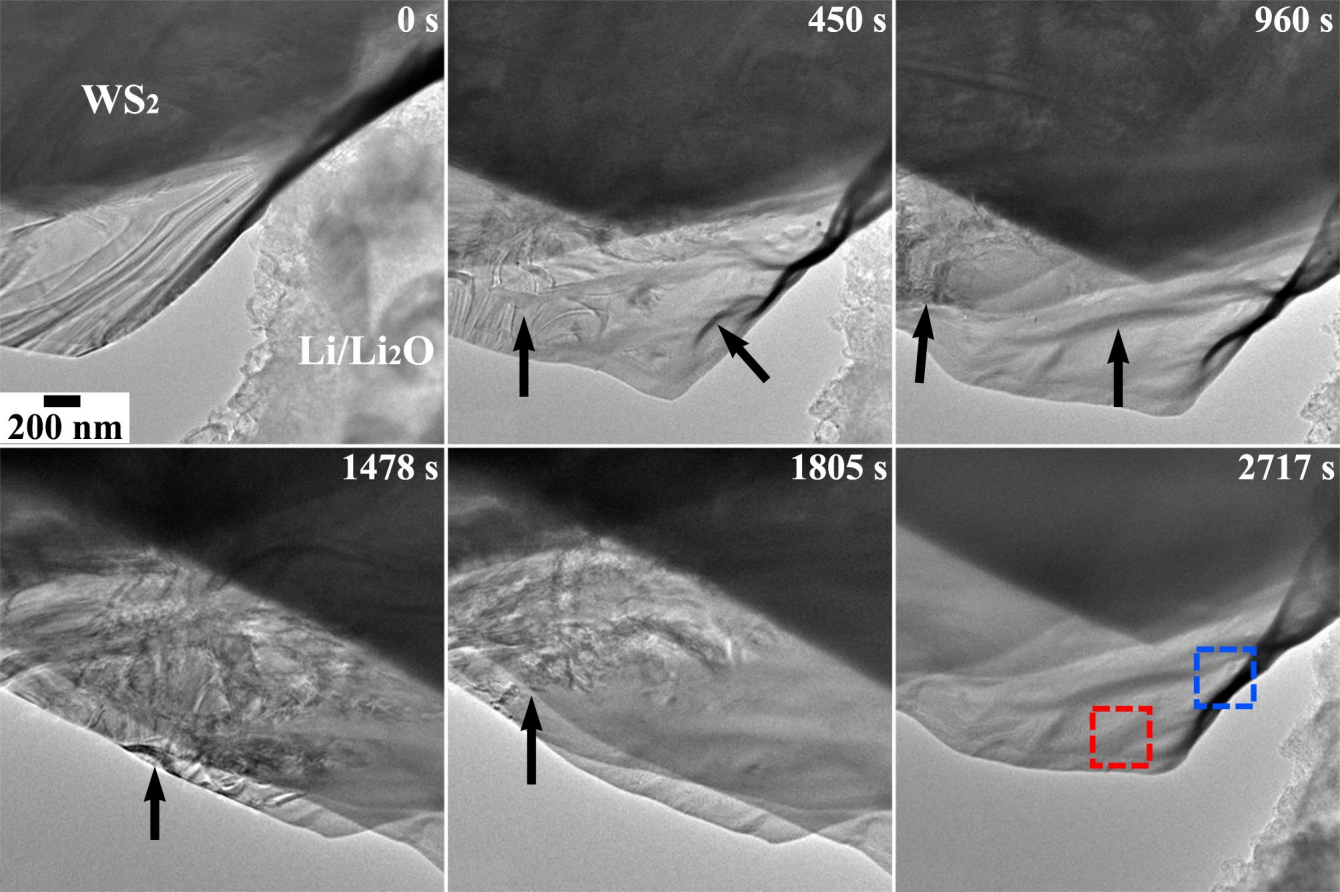
Snapshots at different time frames showing the microstructural changes during Li and WS_2_ reaction. The changes are highlighted with arrows. The colored boxes represent the regions from which the atomic resolution TEM are performed.

Figure 5 shows a series of BF TEM micrographs at different duration (t = 0–2717 s) depicting the progress of the reaction between Li and WS_2_. The entire duration of the video of the Li-intercalation dynamics is given as a supplemental file (S2). The WS_2_ displays a layered contrast before the commencement of the reaction (t = 0 s). As the reaction proceeds further (after 450 s), the contrast arises owing to layering in WS_2_ diminishes near the edge region of the flake and appears to move other part of the specimen as marked with an arrow. Moreover, newer features in the initial reaction domain have also been observed, one of them showing a dark band-like contrast, is highlighted with an arrow. At t = 960 s, the reaction front advances further and a smooth contrast is seen in the previous regions. This trend is continued with further insertion of Li in WS_2_ as shown with arrows in the micrographs at 1478, and 1805s, respectively.

The image at 2717 s displays the initial region of the specimen that is nearly devoid of any contrast. The two squares colored in red and green are portrayed from where atomic scale imaging was carried out and to be presented later. The contrast in the WS_2_ prior to Li insertion can be ascribed to creation of strain during mechanical exfoliation. The observed changes in the contrasts during the intercalation process can be attributed to the localized strains developed during Li intercalation into the WS_2_ interlayers. Subsequently, the diffraction-contrast imaging conditions change and may lead to the appearances and disappearances of imaging features.

**Figure 6:**
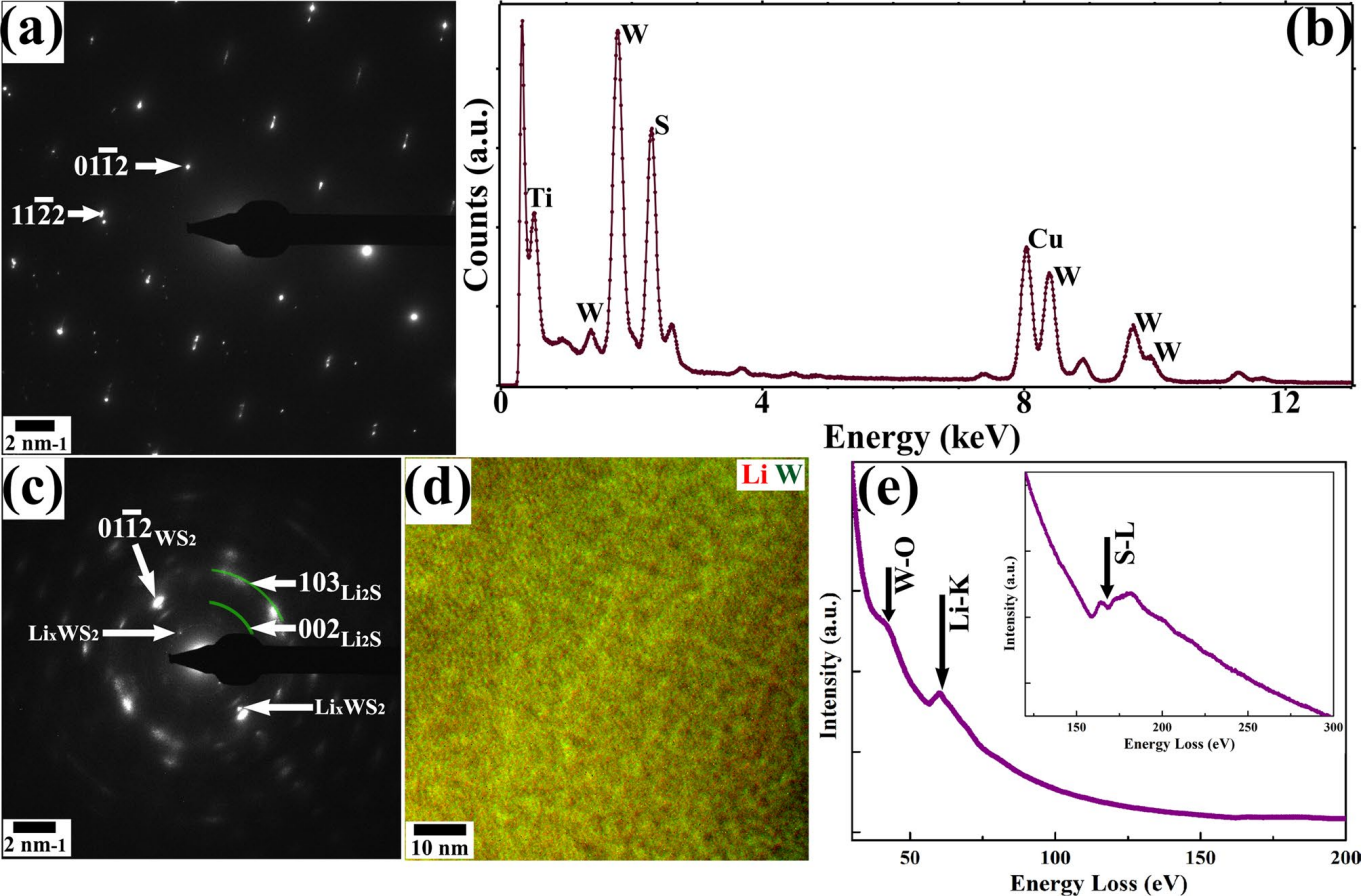
(**a**) SADP indexed as 2H-WS_2_ phase and (**b**) XEDS profile from the pristine WS_2_. Figures (**c**), (**d**), and (**e**) depict SADP, EFTEM map and the EELS profile from the post-lithiated WS_2_, respectively. Coexistence of Li_x_WS_2_ and Li_2_S phases (green arcs) with WS_2_ are shown in the SADP. Energy-filtered TEM map (Li in red, W in green) indicates a possible phase separation reaction. EELS confirms the presence of Li in the reacted specimen. Peaks correspond W-O and S-L energy-loss edges are also shown in the profile.

The SAD pattern and XEDS profile of the pre-lithiated region of WS_2_ are presented in Fig. 6(a,b). The pattern was indexed, and the phase was found to be those of 2H-WS_2_ (ICDD # 00-008-0237). The XEDS clearly shows the peaks of W and S; presence of peaks corresponding to Ti and Cu in the profile is from TEM-STM holder and Cu-grid, respectively. SAD pattern, energy-filtered TEM map, and EELS profile of the post-lithiated specimen from the same area is shown in Fig. 6(c–e), respectively. Superlattice reflections and arcs have been observed in the SAD pattern of the post-lithiated specimen. The analysis revealed the presence of coexisting Li_x_WS_2_ and Li_2_S (ICDD # 01-070-5970) phases (green arcs) in addition to 2H-WS_2_ (cf. Fig. 6c). It is noteworthy here that the reflections corresponding to 2H-WS_2_ are more extended in nature compared to that of the aforementioned pre-lithiated pattern. This is suggestive of the fact that the large grains of pre-reacted 2H-WS_2_ have been fragmented into several crystalline domains with a small angle of misorientation between one another. The energy-filtered image acquired from the post-reacted region exhibits mottled contrast with Li-rich nanometric domains further substantiates the finding of Li_2_S phase from SAD pattern. The EELS profile revealed energy-loss edges corresponding to Li-K (55 eV), W-O (36 eV), and S-L (165 eV) as given in Fig. 6(e). This confirms that intercalation of Li has taken place in the 2H-WS_2_.

**Figure 7:**
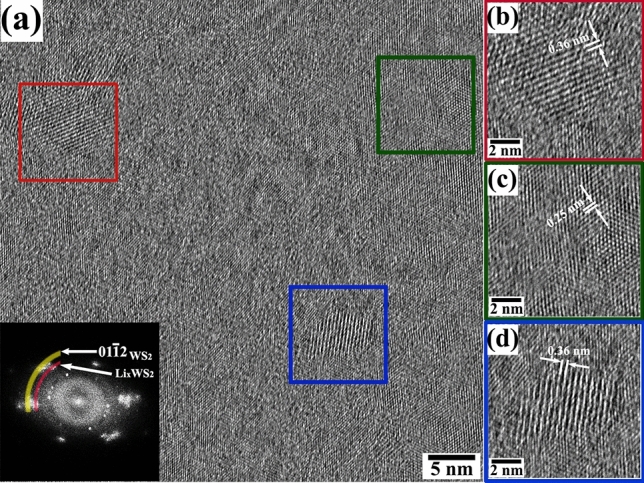
(**a**) Atomic resolution TEM micrograph from the region marked in red square in Fig. 5. Power spectrum (inset) shows the reflections correspond to WS_2_ and Li_x_WS_2_ planes. Figures (**b**), (**c**), and (**d**) depict the magnified views from three different colored boxes marked in (**a**).

As mentioned earlier, the atomic-scale imaging has been performed from the region shown as dotted red square (cf. Fig. 5, 2717 s). Figure 7 shows a representative high-resolution TEM image from the post-lithiated specimen (Fig. 7a) with the power spectrum as inset, accompanied with selected nanocrystalline domains highlighted in red, green and blue squares presented in Fig. 7(b–d), respectively. The smaller crystalline domains of 2H-WS_2_ are observed and correspond to the appearance of less sharp spots (identified by a yellow arc) in the power spectrum. The magnified version of such a region is shown in the Fig. 7(c); the lattice fringe spacing of ~ 0.25 nm corresponds to the 01 $\overline{1 }$ 2 plane spacing of the 2H-WS_2_ phase (ICDD # 00-008-0237). Additionally, two crystalline domains possessing the Li_x_WS_2_ phase are presented in Fig. 7(a,c), respectively. In this case, lattice-fringes spacing are measured to be ~ 0.36 nm and a corresponding long-range periodicity in the power spectrum is marked with the red arc. It is interesting to note that the during Li intercalation, the host matrix is decomposed and lost its long-range periodicity. This nature is further evident from the relatively smaller crystallites size in the post reacted specimen indicating the occurrences of grain fragmentation due to Li intercalation.

**Figure 8:**
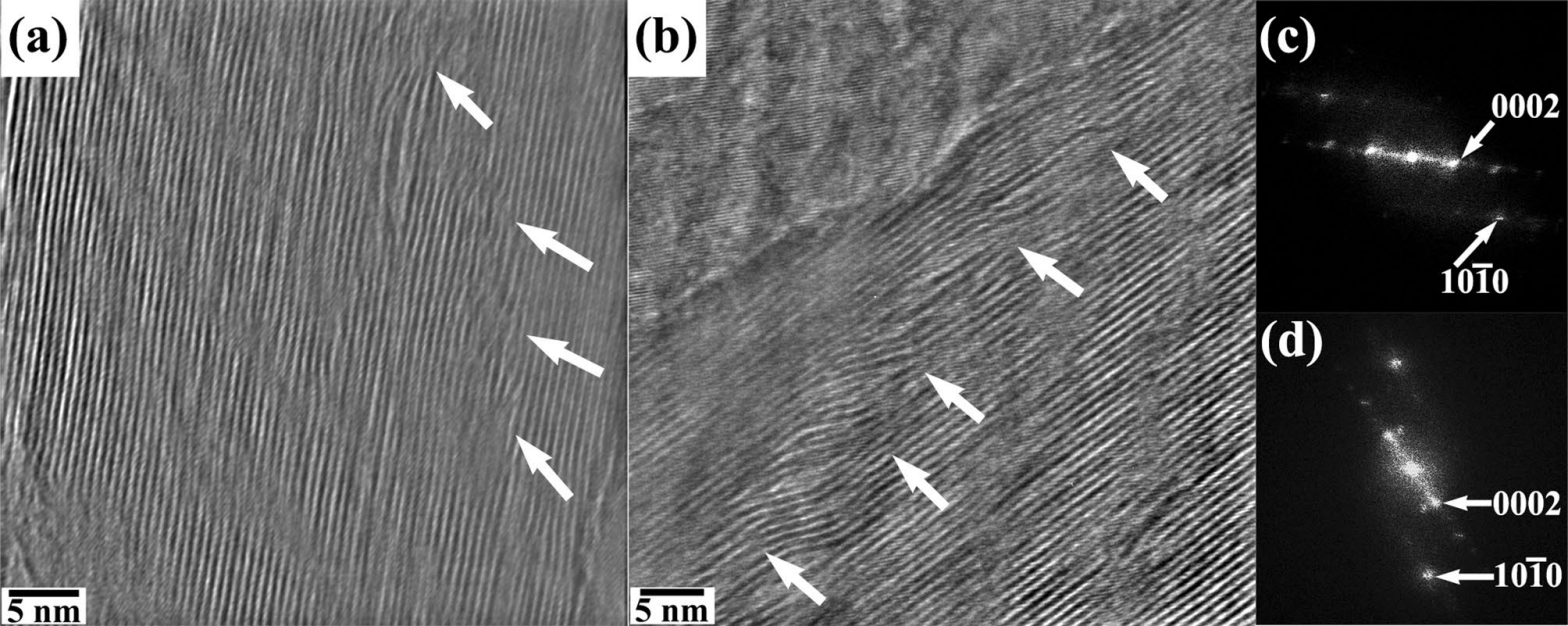
(**a**,**b**) Zero-loss energy filtered atomic resolution TEM micrographs imaged in edge-on condition (region indicated in blue square in Fig. 5) and corresponding power spectra are shown in (**c**,**d**), respectively. The presence of lattice defects are marked with arrows after lithiation.

High-resolution TEM images acquired in edge-on (perpendicular to c-axis) condition from the blue squared (cf. Fig. 5, 2717 s) region of the post-reacted specimen are shown in Fig. 8(a,b), respectively. These micrographs are recorded using the energy-filtered CCD (EFCCD) with a 10-eV energy window around the zero-energy loss peak. During zero-loss imaging only the elastically scattered electrons contribute to the image formation while the inelastically scattered electrons are filtered out. This filtering significantly enhances the image contrast by reducing the background noise. The corresponding power spectra generated from (a–b) are presented in Fig. 8(c,d), respectively. It is interesting to observe the higher concentration of dislocations along 10 $\overline{1 }$ 0 planes. As already reported in earlier works, the presence of the defects can be attributed to the concentration gradient of the intercalating element in the matrix. During intercalation process, Li^+^ channels into the matrix along the interlayer pathway. While intercalating, it is highly possible for the diffusion of Li along the interplanar boundaries through anisotropic diffusion. This leads to the formation of a concentration gradient of Li among the WS_2_ interlayers giving rise to localized Li-rich domains. As a consequence of this, misfit strains developed between the successive interlayers and this relaxes with the formation of line defects^41^.

The in-situ lithiation study of graphite has been presented primarily to (1) compare the observed intercalation results of the TMDs with graphite, and (2) to analyze the intercalation mechanism of Li into the 2D-layered materials. Graphite is structurally the simplest 2D materials where the graphitic layers are stacked together separated by the vdW gaps unlike the TMDs, where the blocks of chalcogen-transition metal–chalcogen are stacked one over the other separated by the vdW gaps. Graphite, having the basic 2D structure, is best suited to represent the non-uniform transformation without posing much challenge from its own structure.

**Figure 9:**
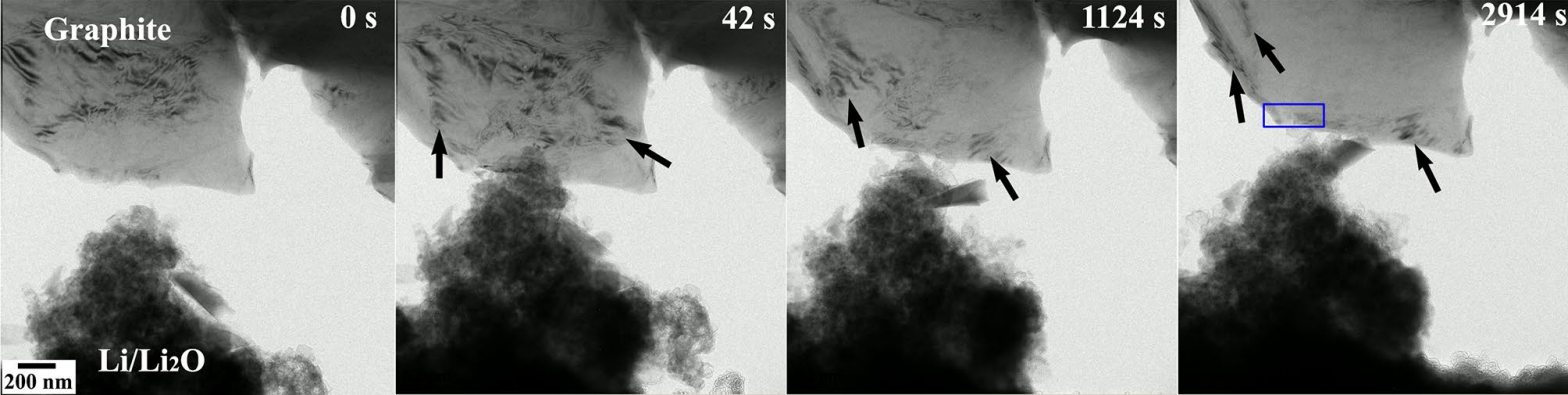
Micrographs depicting the microstructural evolution during Li and graphite reaction. The changes are highlighted with arrows. Blue box indicates the region for further investigation.

Changes in microstructural contrast features along with the structure evolution during in situ solid-state reactions between Li and graphite is shown in Fig. 9. Post-lithiated specimen region considered for further microstructural and microchemical analyses are marked with blue colored box. The associated recorded video has been provided as the supplementary information (S3). The prominent changes in the microstructural features are marked with arrows. As already discussed in the earlier two lithiation processes of MoS_2_ and WS_2_, these contrast changes either arise due to localized strain or due to the changes in the diffraction-contrast imaging conditions resulting from the intercalation of Li in between the graphitic planes.

**Figure 10:**
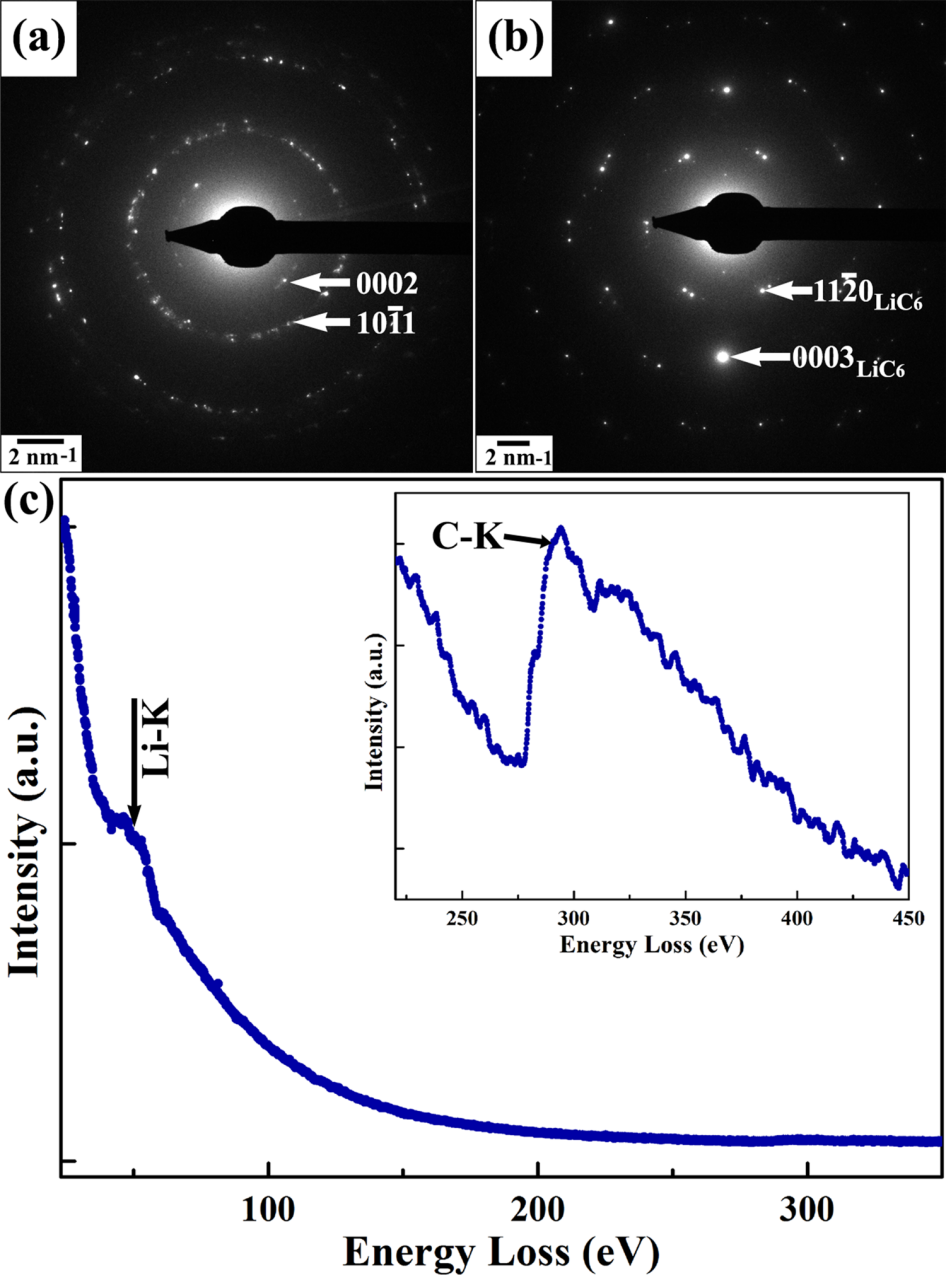
SADPs from the (**a**) pre-lithiated graphite and (**b**) after reaction with Li. SADP indicates the formation of LiC_6_ phase in the post-lithiated specimen. (**c**) EELS shows the presence of Li in the reacted specimen. C-K energy-loss edge after reaction is shown as inset.

The changes in structural and chemical features owing to the Li intercalation in graphite is shown in Fig. 10. Indexed SADPs from the graphite flakes before and after the reaction are displayed in Fig. 10(a,b), respectively. The principal reflections from the post-lithiated system indicate an expansion in the graphitic planes along with the appearance of reflections corresponding to newly formed LiC_x_ structure. It is interesting to note that the SADP in the post-reaction condition eventually becomes more ordered signifying the formation of ordered phases as the reaction products. An electron energy-loss profile from the post-reacted graphite is given in Fig. 10(c) where the energy-loss edges corresponding to Li-K and C-K are marked at 55 eV and 284 eV, respectively. Elemental quantification as performed from the EELS profile confirmed the relative stoichiometry of the post-lithiated product as LiC_5.84_ and can be considered as LiC_6_ for all practical purposes.

**Figure 11:**
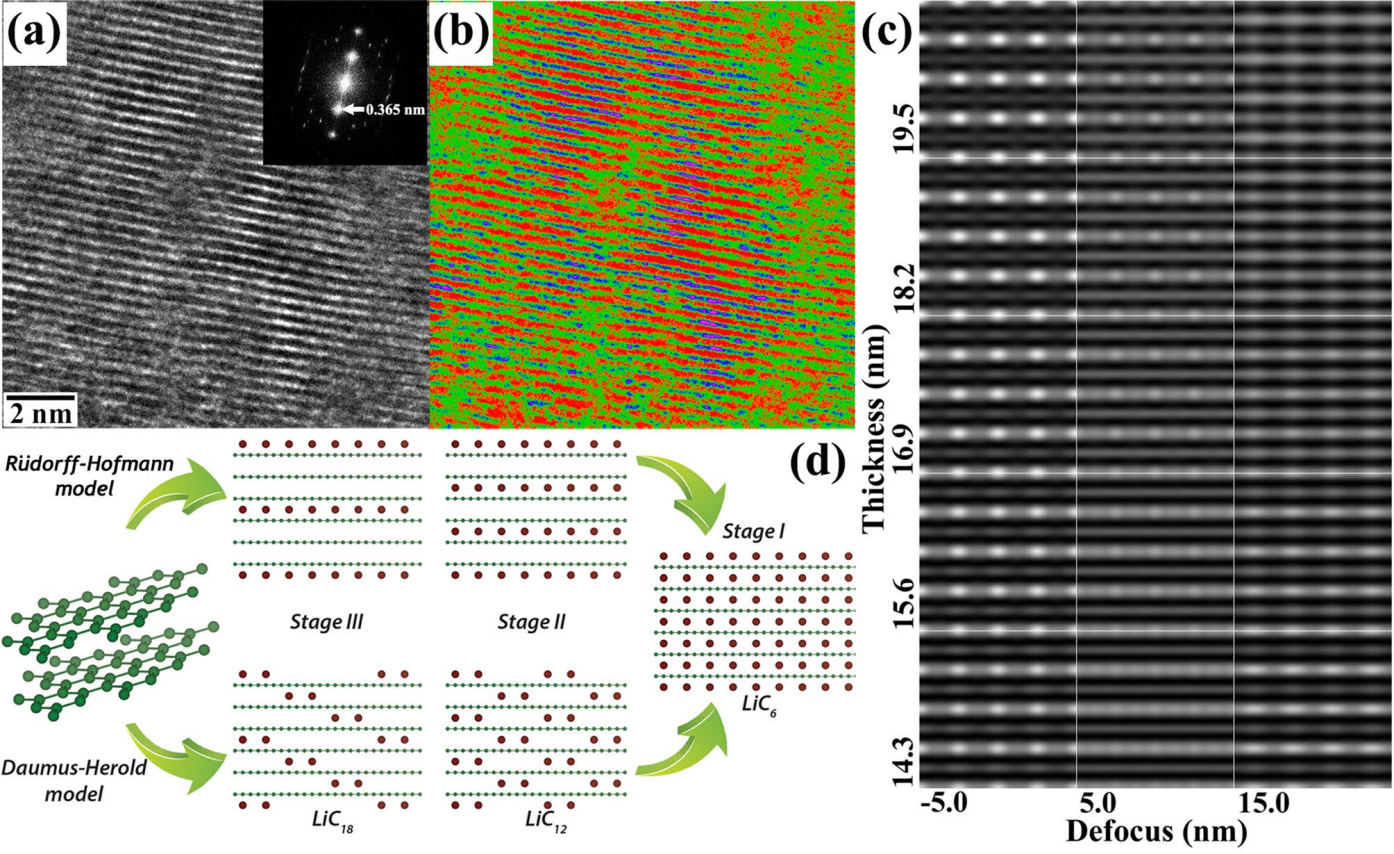
(**a**) Low-dose HRTEM micrograph showing graphitic planes in post-lithiated condition. Inset shows the power spectrum from the same region confirming the lattice expansion along this direction. False colors are inserted for contrast enhancement as displayed in (**b**). (**c**) Thickness-defocus map of LiC_6_ phase along [11 $\overline{2 }$ 0] zone axis using multislice simulation. (**d**) Schematics displaying the Li intercalation mechanisms in graphite according to Rüdorff–Hofmann model and Daumus–Herold model.

The fine structural details of the post-lithiated graphite have been investigated with the low-dose direct-electron detection camera in an image-corrected TEM. The Li intercalation induces interlayer strain into the graphitic planes resulting changes in the localized specimen geometry. This strain makes it possible to image the reacted microstructure in an edge-on condition at several regions of the specimen. Figure 11(a) displays the traces of the 0002 planes in a representative micrograph of the post-lithiated specimen. The power spectrum derived from this region is shown in the inset. The interplanar spacing of the 0002 planes, as measured from the power spectrum, is estimated to be ~ 0.365 nm, which is otherwise reported as ~ 0.335 nm for pristine graphite (ICDD # 00-41-1487). The observed change signifies an expansion of the interlayer spacing which can be attributed to the Li intercalation along graphitic planes. Subtle changes in contrast of the recorded micrograph have been enhanced artificially by false color as shown in Fig. 11(b). It is interesting to observe the color modulation along the graphitic planes (marked in red) which indicates the presence of traces for additional atomic planes in between the van der Waals gaps. Figure 11(c) shows the simulated thickness-defocus (t-∆f) map for the LiC_6_ phase along [11 $\overline{2 }$ 0] zone axis using the multislice algorithm in java-based EMS (j-EMS) platform considering the same imaging parameters in which the aberration-corrected HRTEM image was acquired. The simulated image has been generated for an objective-lens defocus window of − 5 nm to + 15 nm. During the recording of the HRTEM images in the aberration-corrected TEM, the defocus value of the objective lens system was held constant at 0 nm, while the fine-focus control was achieved with a piezo-controlled goniometer-stage movement with nm precision. However, the simulation is performed along a small window ranging − 5 to 15 nm to accommodate any deviation during experimental setup. The thickness variation was considered from 14.3 to 19.5 nm as estimated from the relative thickness measurement (t/λ) during EELS. The schematics of the reported intercalation mechanism of Li in graphite is presented in Fig. 11(d) describing the intercalation pathways in the formation of LiC_6_ phase through different partially lithiated structures.

The lithiation mechanism in graphite has been explained, as noted above, by two different ‘staging’ models, namely the Rüdorff–Hofmann model^69^ and Daumus–Herold model^70^. In the present study, the interlayer intensity (cf. Fig. 11b) indicates that the Daumus–Herold model is relatively more consistent in explaining the lithiation process in graphite because if Rüdorff–Hofmann model is followed, Li has to diffuse within interlayer separation between two stages of partial lithiation. However, none of these existing models can explain the present observation completely and further necessitates the development of the models focused on the localised distribution of Li in the partially lithiated structures rather considering the concentration of the intercalated Li. In the present observation, LiC_6_ forms in small domains indicating that this formation depends on the local intercalate distribution: it involves a few successive layers, and is not controlled by the overall concentration. It is proposed that while forming the lithiated phases, the intercalation of a single Li atom in the graphitic planes facilitates the subsequent intercalation in its nearest neighbor planes, and thus nucleates the embryo of the lithiated structures. Once the nucleus forms it grows by attracting a more of the intercalating atoms from the sparsely lithiated neighborhood to attain the structural stability. This results the formation of intercalated LiC_6_ domains within Li-lean regions. Oswald ripening of the secondary phase then takes place, where the smaller particles dissolves and re-deposited at the surface of the larger particles. In the ripening process, the larger particles are energetically more favored than the smaller particles.

This intercalation mechanism for graphite can then be extended to explain the observed localized phase transformation mechanism in MoS_2_ and WS_2_. In both of these lithiated structures, though the average Li concentration remain constant, localized transformed domains have been observed, depending on the distribution of Li in the adjacent regions.

In contrast to the general concept of phase transformations where the material fully transforms, in the present study, the signature of the localized structural transformation is more prevalent at nanometric domains depending on the distribution of the Li atoms at the interlayers. The lithiation reaction is thus a ‘distribution-dependent’ phenomenon rather a ‘concentration-dependent’ one. This investigation provides evidence of the formation of 2H and 1T structures of phase transformed MoS_2_. However, being a non-kinetically driven process, the time-dependent analysis would only reveal the changes in the phase-transformed domain sizes and not the stabilities of the two coexisting phases.

Moreover, the extent of the voltage-driven diffusion does indeed depend on time because it depends on the current which is carried by the Li ions. What this study shows is that the concentration is not uniform; the structure does not change systematically from one ‘stage’ to the next uniformly across the specimen; thus, the distribution of the different structures must change. The conclusion developed as a result of this study is that the non-uniformity in the concentration is an important factor in how the lithiation proceeds. Subsequently, the current study quantify how the distribution of the regions of different concentration change with time using HRTEM (and the localized frequencies), plus EELS and XEDS. In the case of a diffusion-controlled phenomena, the relative concentration of the intercalated Li increases with the increase in time. The simulated HRTEM images at different Li concentration can thus mimic the microstructural features of the lithiation reaction of MoS_2_ at different reaction time interval. Figure S6 in the supplementary information file shows the simulated HRTEM micrographs of the partially lithiated MoS_2_ phase with different concentration of intercalated Li along [1 0 $\overline{1 }$ 1] zone axis. The changes in the atomic contrast of the partially lithiated MoS_2_ supercell at different Li concentration in figure S6 provides the key to understanding the HRTEM contrast at different time interval during the experiment.

The present analysis of Fig. 11(a–d) also invokes an intriguing discussion over the feasibility of imaging light atoms in HRTEM. Detection and imaging of the Li in TEM is a perennial problem since under the electron radiation, light atoms no longer remain stationary. This problem is more evident in the case of STEM-HAADF spectral imaging (SI) where the very small probe contains produces a high electron dose resulting beam damage and deformation in the specimen. Energy-filtered TEM (EFTEM) is another viable method for the imaging of Li, but in all practical situation the higher-order plasmon energy-loss edges overlaps with the pre-edge of Li-K edge, thus creating additional challenges in both the three window and two window methods of EFTEM analysis. The situation become even more challenging, when the relative concentration of the intercalating Li is significantly lower and has not yet resulted in interstitial ordering. A few reports are available on the imaging of the light elements using annular-bright field (ABF) TEM^74^ and also using the integrated differential phase-contrast (iDPC) technique^75^. More recently, cryo-TEM has gained popularity for imaging the low atomic-number elements in the battery materials^76,77^. In the present study, to minimize the beam effect, atomic-resolution phase-contrast imaging has been performed in an image aberration-corrected TEM using a low-dose direct-electron-detection camera. This is arguably one of the most promising phase-contrast imaging modes in the TEM for probing the structural details of the intercalated phases; the processed image (cf. Fig. 11(b)) also indicates contrast between the graphitic planes. It is noteworthy to mention that in the simulated map also Li and C both are providing visible contrast in the present imaging conditions supporting the possibility that the contrast seen between the graphitic planes could be the traces of Li.

The lithiation of 2H-MoS_2_, 2H-WS_2_, and graphite leads to structural modifications in each one even though the nature of the observed phase change is different. The reaction equations of Li with MoS_2_, WS_2_, and graphite stated as follows^78–80^,

$$\begin{gathered} {\text{MoS}}_{{2}} + x{\text{Li}}^{ + } + x{\text{e}}^{ - } \to {\text{Li}}_{x} {\text{MoS}}_{{2}} , \,0{ \le }x{ \le }{1} \hfill \\ {\text{WS}}_{{2}} + x{\text{Li}}^{ + } + x{\text{e}}^{ - } \to {\text{Li}}_{x} {\text{WS}}_{{2}} , \,0{ \le }x{ \le }{1} \hfill \\ {\text{C}}_{{6}} + {\text{ Li}}^{ + } + {\text{ e}}^{ - } \to {\text{ LiC}}_{{6}} \hfill \\ \end{gathered}$$

With the inclusion of more Li ions, the partially lithiated Li_x_MoS_2_ and Li_x_WS_2_ phases will convert into Mo/W and Li_2_S as per the following conversion reactions,

$$\begin{gathered} {\text{Li}}_{x} {\text{MoS}}_{{2}} + \, \left( {{4 } - x} \right){\text{Li}}^{ + } + \, \left( {{4 }{-}x} \right){\text{e}}^{ - } \to {\text{Mo }} + {\text{ 2Li}}_{{2}} {\text{S}}, {1} \le x \le {4} \hfill \\ {\text{Li}}_{x}{\text{WS}}_{{2}} + \, \left( {{4 } - x} \right){\text{Li}}^{ + } + \, \left( {{4 }{-}x} \right){\text{e}}^{ - } \to {\text{W }} + {\text{ 2Li}}_{{2}} {\text{S}}, {1} \le x \le {4} \hfill \\ \end{gathered}$$

In corroboration with these equations, present work exhibits that MoS_2_ undergoes a local phase transformation from 2H to 1T-Li_x_MoS_2_ that then coexists with the untransformed 2H-MoS_2_. Orthorhombic Li_2_S (o-Li_2_S; ICDD # 01-070-5970) has also been observed (cf. green arcs in Fig. 3a). These findings are consistent with existing reports^65,66,81^. Similar structures are also observed in 2H-WS_2_, i.e., 1T-Li_x_WS_2_ and o-Li_2_S in the post-lithiated specimen. However, a distinct microstructural change has been seen in WS_2_ after Li intercalation; the initially extended grains present in pre-reacted specimen became fragmented during lithiation. This change is evidenced by the appearance of streaking in the principal reflections of 2H-MoS_2_ (viz. the 01 $\overline{1 }$ 2 reflection) and in the greater number of reflections corresponding to the o-Li_2_S phase in the SADP of post-lithiated WS_2_. Additionally, HRTEM analysis from both of the post-lithiated specimen of MoS_2_ and WS_2_ confirms the coexistence 2H and 1T structure rather than complete transformation into the 1T phase.

This dual-phase lithiated structures are supported by the DFT calculations; it is reported that the energy of the hybrid 1T/2H structure is lower than individually lithiated 2H or 1T phase and can be attributed to the stronger Li binding in the hybrid phase at the interface^58^. However, the domain size of the 1T phase in the lithiated MoS_2_ and WS_2_ varies depending on the concentration of the intercalated Li in these systems. For monolayer MoS_2_, the 2H to 1T structural transformation requires a Li concentration of 30–40%^47,49^. However, the same is not applicable for the bulk MoS_2_ with multiple layers, where the triggering of the 2H to 1T phase transformation does not require any critical concentration of Li: after the intercalation of two consecutive layers of 2H structure, it is likely that the MoS_2_ layer sandwiched between two Li layers transforms to the 1T phase^58^. The number of the successive lithiated interlayers determines the domain size of the transformed 1T phase and critically depends on the Li concentration in the intercalated structures.

## Conclusions

The following conclusions can be drawn from the present study,

1. Phase transformation and structural modification takes place during in situ reaction between Li and 2D materials (MoS_2_, WS_2_ and graphite) inside TEM. Formation of Li_x_MoS_2_ and Li_x_WS_2_ phases are confirmed along with Li_2_S in the lithiated MoS_2_ and WS_2_. In post-lithiated graphite, domains of LiC_6_ has been found along with an increase in interplanar distances.
2. EELS studies inferred the difference in the relative concentration of Li in the intercalated MoS_2_, WS_2_ and graphite flakes. This can be attributed to the difference in the binding energy of Li with the different 2D systems under consideration. In lithiated WS_2_, possible signature of elemental partitioning is observed in EFTEM analysis.
3. A dual-phase microstructure consisting of domains of 2H and 1T phases is observed in both MoS_2_ and WS_2_ after Li intercalation. The structures are analyzed with HRTEM image simulation using DFT-optimized crystal information files. However, the domain size varies significantly from lithiated MoS_2_ to WS_2_. In post-lithiated WS_2_, the 1T and 2H domains are smaller with an indication of grain fragmentation.
4. Two models have long been considered to explain the intercalation mechanism but there has never been a direct observation that could differentiate between the two. Present study shows that the process is actually fundamentally different from either of these models and that regions of fully lithiated material can develop locally and that these can grow by a ripening process confirming the reaction as a ‘distribution-dependent’ phenomenon rather a ‘concentration-dependent’ one.
5. Imaging Li in lithiated graphite is, in principle, feasible with the aid of low-dose imaging in aberration-corrected TEM. Possible contrast from the intercalated Li between the graphitic sheets has been detected by combining image processing and simulation. The current approach can be adapted for the imaging of other light elements in the vicinity of high atomic-number elements.

## Materials and methods

The flakes of the 2D layered materials considered in the present study are mechanically exfoliated from the respective single crystalline bulk samples of MoS_2_, WS_2_, and graphite using the scotch-tape method. The exfoliated flakes are then dispersed in acetone and collected on a Cu half-grid of 400 mesh.

The in situ reaction inside the TEM has been carried out using a Nanofactory Instruments TEM-STM holder. The detailed design of the electrochemical cell in this holder has been reported earlier^22,40^. In brief, (1) the Cu half grid containing the exfoliated nanoflakes are affixed with a fine Al wire using conducting epoxy and subsequently screwed to the top input of the TEM-STM holder. (2) the other end of the holder is fitted with a removable, six-legged socket called a “top-hat” that consists of mechanical, piezo-driven movement controllers for coarse and fine controls with nm precision. The Li metal is scratched with an electro-chemically sharpened Tungsten (W) wire and screwed with the top-hat onto the other end of the holder.

The entire Li handling process is been performed inside an Ar-filled glovebox. Once the holder is loaded with the exfoliated samples and the Li metal, it is quickly transferred to the TEM. However, due to the atmospheric exposure during the transfer process, the Li metal may be partially oxidized and a mixture of Li/Li_2_O considered as the solid-state electrolytes for all practical purposes.

In situ reactions between Li and the layered materials (MoS_2_, WS_2_, graphite) were performed inside the Tecnai G2 F30 TEM operated at 300 kV. During in situ lithiation of MoS_2_, an external electrical bias of − 2 V has been applied using the Nanofactory Instruments bias control. During the lithiation of WS_2_ and graphite, the external biasing voltage was varied in the range between – 2 and − 4 V and reactions were allowed to continue until any visible microstructural changes appeared to cease. The microchemical characterization of the pristine and the lithiated specimen were carried out using X-ray energy dispersive spectroscopy (XEDS), Electron energy-loss spectroscopy (EELS) and Energy-filtered TEM (EFTEM). HRTEM investigations of the post-lithiated specimen have been carried out in a Titan image aberration-corrected Environmental TEM (ETEM) using a direct-electron detection, low-dose (10.569 e^−^/px/s) camera (Ametek/Gatan K3-IS) to minimize the electron beam damage to the sample. All the first-principles calculations are performed within the framework of DFT calculations using the Vienna Ab-initio Simulation Package (VASP)^82^. The ion/electron interactions are derived using the projector augmented-wave (PAW) method^83^ and the generalized gradient approximation formulations by Perdew–Burke–Ernzerhof (GGA-PBE)^84^ was used to express electronic exchange correlations. Atomic resolution phase contrast image simulations are carried using the multislice algorithm within the java-EMS (j-EMS) program^85^.

## Supplementary Information

Supplementary Information 1.

Supplementary Video 1.

Supplementary Video 2.

Supplementary Video 3.

## Data Availability

The datasets generated during and/or analysed during the current study are available from the corresponding author on reasonable request.
