# Supplementary material for: Phase evolution and structural modulation during in situ lithiation of MoS2, WS2 and graphite in TEM
Source: Sci Rep. 2021 Apr 27;11:9014. doi: 10.1038/s41598-021-88395-1 (PMC8079398; doi:10.1038/s41598-021-88395-1)
Supplement: Supplementary file 1 — Supplementary Information 1. [file 41598_2021_88395_MOESM1_ESM.docx]

# Phase Evolution and Structural Modulation During *in situ* Lithiation of MoS_2_, WS_2_ and Graphite in TEM

Chanchal Ghosh^*†1^, Manish Kumar Singh^*†1^, Shayani Parida^1^, Matthew T. Janish^1^, Arthur Dobley^2^, Avinash M. Dongare^1^, C. Barry Carter^3,4^

^1^Department of Materials Science and Engineering, University of Connecticut, Storrs, CT-06269, USA

^2^EaglePicher Technologies, East Greenwich, RI-02818, USA

^3^Department of Chemical & Biomolecular Engineering, University of Connecticut, Storrs, CT-06269, USA

^4^Center for Integrated Nanotechnologies (CINT), Sandia National Laboratories, Albuquerque, NM-87185, USA

*Corresponding Authors email: [chanchal.ghosh@uconn.edu](mailto:chanchal.ghosh@uconn.edu); [manish.singh@uconn.edu](mailto:manish.singh@uconn.edu);

† Authors with equal contribution

**Supplementary Materials:**

Videos corresponding to the *in situ* lithiation of MoS_2_, WS_2_ and graphite are provided as S1, S2, and S3, respectively in supplementary material. The videos show the microstructural transformation in MoS_2_, WS_2_ and graphite during solid-state reaction with Li in real space at *real time* inside the TEM. The video speed in all three cases has been digitally increased from the originally recorded ones.

Details of the indexing scheme for the SADP (cf. Figure 1(a)) using the Miller-Bravais indices is provided in S4. Figure S4(a) shows the SADP from the pristine MoS_2_, where two diffraction spots (marked as A and B) are considered for further analysis of the pattern and also to determine the zone axis. The interplanar spacing correspond to A and B spots are measured as 0.267 nm and 0.250 nm. The interplanar angle between these two spots are measured as 59°. Based on these measurements, the Miller indices for these two spots are determined as (1 $\bar{1}$ $\bar{1}$) and (1 0 $\bar{2}$) respectively. In Miller-Bravais system, these two spots are indexed as (1 $\bar{1}$ 0 $\bar{1}$ ) and (1 0 $\bar{1}$ $\bar{2}$) respectively. Zone axis of this SADP, as determined in three index system through the vector cross-product of (1 $\bar{1}$ $\bar{1}$) and (1 0 $\bar{2}$) planes results as [2 1 1]. The zone axis in the Miller-Bravais index system can be determined from the Miller index system using a hexagonal transformation ^1,2^ and can be written in the following way,

U = (2u-v)/3

V = (2v-u)/3

T = -(u+v)/3

W = w

Where, [U V T W] is the zone axis in Miller-Bravais index system and [u v w] is the zone axis in Miller index system. Using the above transformation equations, [2 1 1] zone axis in Miller index system can be written as [1 0 $\bar{1}$ 1] in the Miller-Bravais system.

For further confirmation, the SADP for the pristine MoS_2_ along the experimentally measured zone axis has been simulated using j-EMS software and is displayed as Figure S4(b).

**
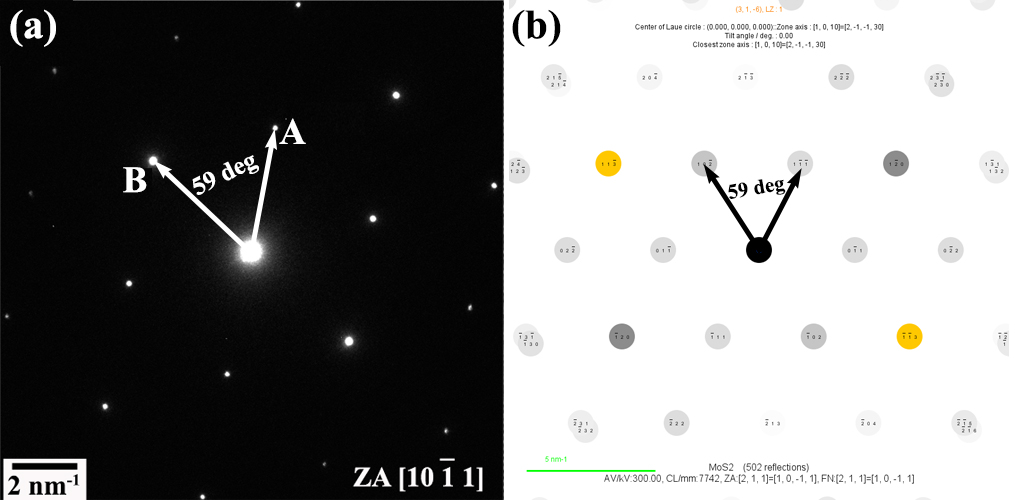
**

***Figure S4:*** *(a) SADP from pristine MoS_2_. (b) Simulated SADP from MoS_2_ along the same zone axis.*

| 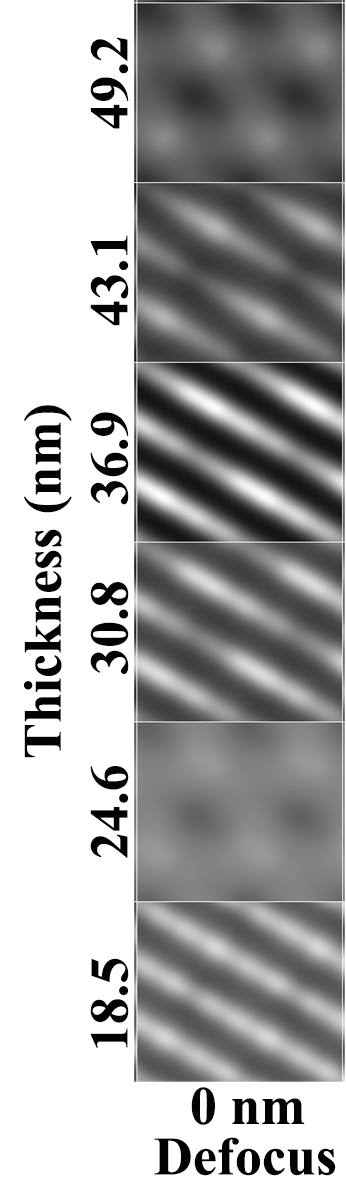 | ***Figure S5:*** *Thickness-defocus map for partially lithiated hybrid 1Li-MoS_2_ structure.* |
| --- | --- |

Figure S5 displays the simulated thickness-defocus map generated from the DFT optimized structure of partially lithiated hybrid 1Li-MoS_2_ phase. The simulation has been carried out at the aberration-corrected TEM conditions with 0 nm defocus and for a thickness range of 18 nm to 50 nm. Figure S5 confirms even with 0.1 relative concentration of Li atoms in MoS_2_, individual atomic contrast is no longer visible in the simulated HRTEM image

**Reference:**

1 Edington, J. W. *Electron Diffraction in the Electron Microscope* 1-77 (Springer, 1975).

2 Frank, F. On Miller–Bravais indices and four-dimensional vectors. *Acta Crystallographica* **18**, 862-866 (1965).

In case of a diffusion-controlled phenomena, the relative concentration of the intercalated Li increases with the increase in time. The simulated HRTEM images at different Li concentration can thus mimic the microstructural features of the lithiation reaction of MoS_2_ at different reaction time interval. Figure S6 shows the simulated HRTEM micrographs of the partially lithiated MoS_2_ phase with different concentration of intercalated Li along [1 0 $\bar{1}$ 1] zone axis. The structures of the partially lithiated MoS_2_ phase are determined using DFT modeling. Details of the DFT modeling and the HRTEM image simulation has been provided in the Materials and methods section of the paper. The changes in the atomic contrast of the partially lithiated MoS_2_ supercell at different Li concentration provides the clue of the HRTEM contrast at different time interval during the experiment. The lattice contrast changes from linear to discreate with the increase in Li concentration (1Li to 5Li) and subsequently tends to be linear again for 9 Li-MoS_2_ supercells.


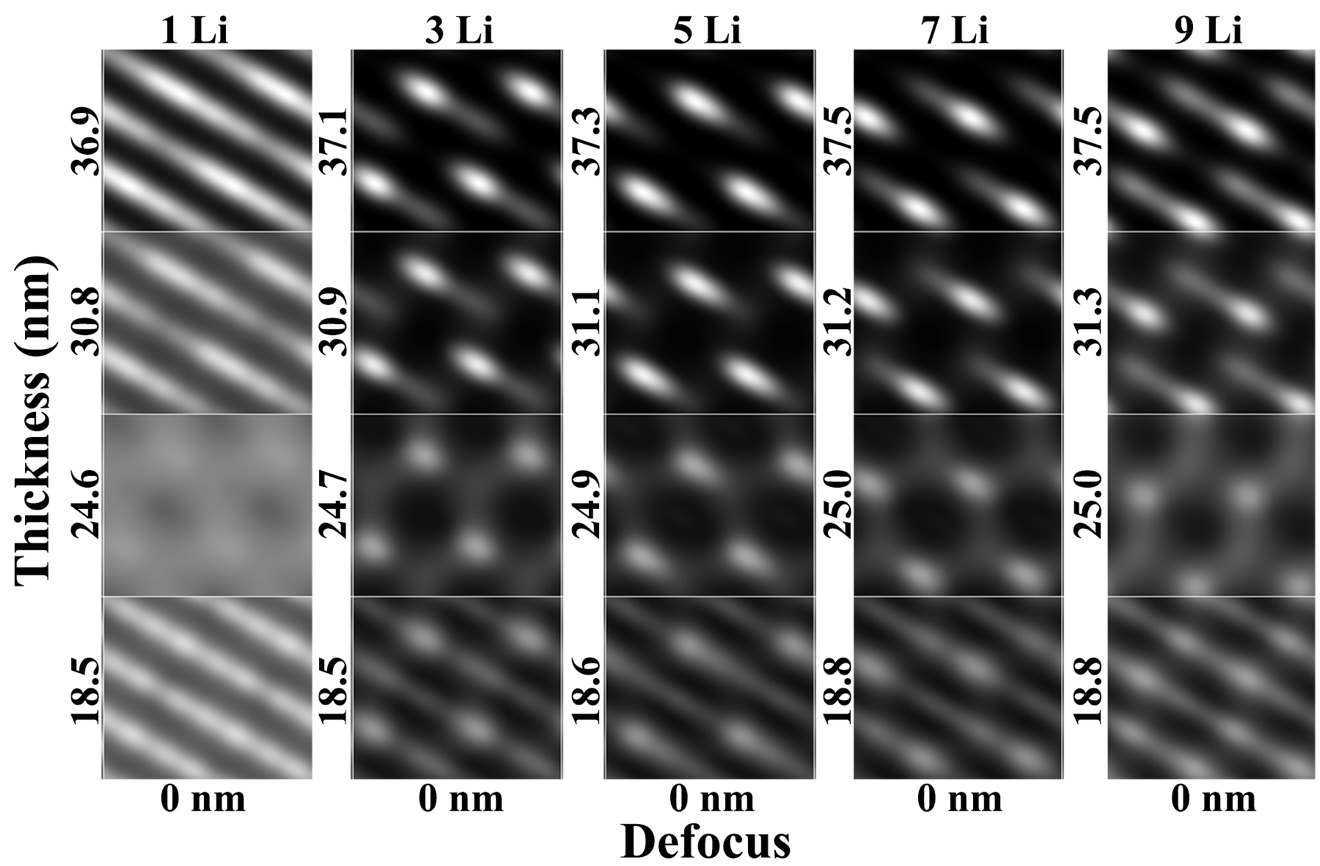


***Figure S6:*** *Simulated HRTEM micrographs of partially lithiated MoS2 phase with different concentration of intercalated Li.*
